# 

**Published:** 1891-01

**Authors:** 


### INDEX TO VOLUME XLV.

COMMUNICATIONS.

Artificial Tooth Crowns, J. B. Carmichael, D.D.S.......... 57
Atomic Weights, W. H. Whitslar, D.D.S............ 165
American Dental Association, Thirty-First Annual Meeting, N. S. Hoff, D.D.S........................ 525
American Medical Association, Section of Oral and Dental Surgery.................... 559
Atkinson, Dr. W. H.............. 388
A Dentist and La Grippe, C. M. Wright, D.D.S.......... 289
Brain and Fingers................ 167
Bromide as a Disinfectant..... 490
Chloralamid in Surgery, Emory Lanphear, M.A., M.D.............................. 435
Chloride of Methyl, L. E. Custer, D.D.S.................... 20
Conducting Dental Societies, General Manner, Dr. J. W. Lyder......................... 70
Case of Cancrum Oris, Report W. P. McDonald, M.D.... 161
Contour Filling, The Genesis of, by Geo. S. Allen, D.D.S. 577
Dental Ethics, W. A. Knowles, M.D., D.D.S................. 555
Dreams and Visions............. 27
Diseases of the Teeth and Their Relation to the General Health, W. M. Williams, M.D............. 72
Development of Teeth, W. H. Whitslar, M.D., D.D.S.... 270
Disease of the Antrum of Highmore, Leopold Neuman, D.D.S.................... 549
Experimental Implantation of Teeth, Notes on, M. H. Fletcher, D.D.S., M.D..... 5
Evolution of Dentistry, J. A. Robinson, D.D.S............ 124
Eye to Business, .................. 246
Hypnotism, by J. R. Callahan, D.D.S.................... 31
Health of the Dentist, by Howard Van Antwerp, D.D.S.......................... 119
Hygiene of the Mouth, by J. F. Dougherty, D.D.S....... 335
History of Dentistry as a Coordinate Branch of the Healing Art, by F. S. Whitslar....................... 265
Investigation of the Cranium and Jaws, Scientific, by Eugene S. Talbot, M.D., D.D.S......................213, 473
Ideal College Curriculum, by Milton F. Ault, M.D., D.D.S.............................. 421
Iron and Manganese, Biological Action.................... 437
Lecture and the Lesson, by C. M. Wright, D.D.S... 285
Latest Theories of Dental Caries, by Dr. Calhoon.... 320
Mouth Breathing not the Cause of Contracted Jaws and High Vaults, by Eugene S. Talbot, M.D., D.D.S. 594
Michigan Dental Law, .......... 617
Nitrogen a Necessity in the Development of the Teeth, by W. H. Whitslar, M.D., D.D.S.............................. 386
New Antiseptic in Dentistry A, by W. H. Whitslar, M.D., D.D.S.................. 163
Ohio State Dental Society, Discussion of Dr. Custer's Paper.......................... 24
Oldest Dental Book, The Translation................... 369
Phagocytosis in Dental Lesions, by F. W. Sage, D.D.S...........................220
Preparation of Cavities and How to Fill Them, by Dr. D. R. Jennings.............. 317
Preparing Sections of Teeth for Histology and Bacteriology, by Vida A. Latham........................276, 323

Possibilities of Medicine, The 390
President's Address, by M. H. Fletcher 482
Recurrence of Decay, by J. G. Templeton 281, 283
Relations of Bacteria to Practical Surgery, by John S. Roberts, M.D 235
Rubber Dam, Means of Holding While Operating upon Cavities in Labial and Buccal Surfaces, by C. R. Butler 36
Salivary Calculous, Origin and Cause of Accumulation on the Teeth, by W. Williams, M.D 114
Stomatitis, Translation by Dr. Marie Pierre 291
Stomatitis — Especially with Reference to Dentition and Dental Surgery, by V. A. Latham 605
Starved Teeth 488
Teeth of Invertebrate Animals, by Alton H. Thompson, D.D.S 587
Tumor, Five Cases of Removal of the Jaw for, by W. D. Hamilton, M.D 63
Tumor, Aneurismal, Treated by Injection, by John S. Marshall, M.D 109
Temperance Proverb 123
Why Copper Amalgam sometimes Wastes in the Mouth, by W. B. Ames, D.D.S 226
What Circumstances and Conditions Modify the Action of Solvents? by F. W. Fleming 116
"Your Old Men Shall Dream Dreams, and Your Young Men Shall See Visions," by N. S. Hoff, D.D.S 27

PROCEEDINGS.

Alumni Association of the Philadelphia Dental College 502
Forty-Seventh Annual Meeting of the Mississippi Valley Association of Dental Surgeons 247
Missouri State Dental Ass'n 620
National Association of Dental Faculties 491
National Association of Dental Examiners 498
Odontograph Society, Pittsburgh, Pa 203
The Northern Ohio Dental Association 341
Thirty-First Annual Meeting of the American Dental Association 438

SELECTIONS.

Ambidexterity 82
An Animated Manikin 185
An Anxious Doctor 346
Artificial Quinine 346
Advertising — "Private and Confidential" 511
Anæsthetics—Statistics of 453
Adulteration Laws in Russia. 180
Bacterial Poisons 505
Bacteriology--Cardinal Points in 412
Bacteriology — Run to Extremes 45
Bone Grafting 99
Blood Supply of Nerves 175
Biglow, Dr. Henry J., and the Discovery of Anæsthesia 183
Bacillary Partnerships 197
Bromide as a Disinfectant 490
Baltimore Letter to Journal of American Medical Association Extract 407
Bromide of Ethyl in Dental Practice 452
Brain Rest 411
Bathing, Rules for 513
Cold in the Head 179
Cocaine, Physiological Action and Uses 40
Cocaine Injections—Death in a Dentist's Chair from 149
Cocaine— Action on the Circulation 403
Cocaine Incompatibles 351
Cocaine Again 195
Clinical Lecture — Selections from 94
Chloroform and Ether — Action of 96
Comparative Anatomy, Study of, by A. H. Thompson, D.D.S 129

Curve of Health, .................. 144
Coffee.............................. 193
Concentrated Foods............. 194
Constituents of food.............. 302
Centenary of the Microscope... 348
Cystoma of Jaw................... 405
Campho-Phenique............... 410
Cough Mixture, A Good........ 510
Caries, Dental..................... 510
Camphorated Naphthol ........ 90
Chilblains.......................... 98
Chloroform iu Convulsions.... 128
Cod Liver Oil, Disguise for.... 152
Diphtheria, Treatment of....... 95
Diet in Diphtheria with Special Consideration of the Proper Food for Children 84
Diet, the Relation of to Personal Beauty................. 80
Dental Protective Association of the United States, The Second Annual Meeting... 100
Dental Protective Association of the United States, Communication from........... 306
Deafness for High Notes........ 143
Disinfection of Hands........... 177
Dyspepsia, Causes of............ 185
Deciduo-Permanent Teeth..... 186
Dietetic Progress................. 301
Defective Teeth.................... 193
Drinking Ether..................... 458
Doctor, the Old Family.......... 463
Dysentery, Treatment of by Irrigation of Lower Bowels................................ 187
Ethics of Experimentation upon Living Animals, The.............................. 170
Epistaxis, Simple Method for Controlling.................... 410
Embryology ........................ 90
Eczema of Dentition, Solution for the.......... ........ 184
Electricity, Absorption of Tumors by......................... 188
Feeding the Young, by O. H. Phelps, M.D.................. 84
Food Products and Their Chief Adulterants........... 305
Face, How to Wash,............ 362
Fire, How to Extinguish....... 461
Florda's University.............. 188
Harvard Medical School, Four Years' Course at, The...... 334
Hippocrates on Injuries to the Skull, Their Surgical Treatment..................... 454
How to Clean Old Slides and Utilize Spoiled Mounts... 451
Hypodermic Syringe ........... 47
Health Commandments, The Ten.............................. 91
Hot Water Remedies............ 154
Hot Water for Sleeplessness... 140
Hypnotism in Public Forbidden.............................. 301
Hypnotic—Somnal, The New 141
Hints for the Profession......... 396
Heredity, Some Reflections on it................................. 394
" In Simplici Salus "........... 403
Items Worth Reading........... 407
Inebriety and Medical Practice................................ 509
Iodoform, Deodorization of by Creolin......................... 361
Items................................ 186
Italian Quacks and Lymph... 187
Influence of Organic Acids upon the Salivary Conversion of Starch............ 191
Infantile Convulsions........... 191
Iodoform, Camphor a Solvent for.................................. 39
Iron and Manganese, Biological Action of................ 437
Iodoform Odor, to Remove from the Hands............. 453
Koch, Dr........................... 138
Koch's Lymph, The Composition of......................... 139
Kochism Denounced by Dr. Virchow....................... 334
Koch's Cure for Consumption 189
Legalized, The Metric System 513
Laryngological Curiosity....... 151
Lukewarm Baths to Restore the Warming Powers of Age to Youthful Vigor... 461
Liniment for Bruises........... 507
Longest Medical Courses....... 149
Microscope Lenses, Improvement in......................... 460
Method of Differentiating Nerve Tissue with Mercuric Chloride............... 504
Meetings.................363, 364, 368

Method of Changing Bodily form by the Administration of Certain Foods Suggestions of .............. 89
Micro-Organisms of Cancer.... 393
Micro-Organisms in Clothing. 153
Micro-Organisms in the Human Mouth.................. 197
Microbes in the S omach ...... 189
Mummies ........................ 303
Measuring the Perception of Odor.......................... 351
Mouth of the Infant at Birth.. 408
Man, A Deciduous............... 150
Monkey Solves the Problem 462
Medical Practice in Minnesota 148
Medical Achievement in China 187
Nitrous Oxide, Therapeutic Application of.............. 147
Nasal Obstructions and Spinal Deformities.......... ........ 153
Neuralgia of the Head, Cures for.............................. 305
Not so Bad......................... 385
Names of the Grip.............. 401
New Disease........................ 411
New Anæsthetic,................. 503
Nitrite of Amyl in Chloroform Poisoning, by E. Mammen, M.D...................... 509
Oliver Wendell Holmes, "De Senectute"..................... 49
Obesity, Treatment of........... 145
Obesity, Simple Method of Curing......................... 459
Oatmeal.............................. 303
Odontological Society of England — "Ready Contours and Crowns Made of Amalgam"................... 295
Overworked Physician's Luncheon,........................ 148
Pyoctanine.......................... 190
Preventing Infection of Operative Wounds, Best Method for.................. 96
Patent Rights........................ 171
Progress of Surgery in 1890... 178
Primary Dentition, Management of........................ 181
Prophylaxis......................... 182
Pathlogy of Grief................. 462
Peroxide of Hydrogen........... 250
Pathogenic Bacteria, Products of ...................................... 142
Peroxide of Hydrogen and Ozone, Their Antiseptic Properties..................... 76
Phosphate of Lime.............. 189
Re-Union of Cut Off Tongue.. 97
Rights and Lefts.................. 304
Rubber Dam, The Use.......... 397
Syphilis, Transmissibility of.. 456
Syphilis.............................. 508
Syphilis, Relation of to the Practice of Dentistry...... 42
Sound Body First, A............. 44
Sterilized Instruments, — To Save the Edge of............ 50
Steno's Ducts—A Case of Occlusion.......................... 93
Surgical Shock.................... 151
Shock During Operations under Anæsthetics, Question of................................ 98
Sanity and Insanity............. 154
Specialism, Dr. Holmes on..... 168
Spontaneous Generation........ 176
Sir Richard Quain............... 194
Salt in Milk for Children...... 452
Strychnine, Effects on the Stomach........................ 460
Starved Teeth...................... 488
"So Much a Foot".............. 150
Solubility of New Medicines.. 192
Sweating of the Hands.......... 453
Tea Drinking and Cold Feet.. 152
Tennessee Dental Law.......... 446
Toothache Drops.................. 451
Transplantation of Teeth...... 455
Teeth in Diabetes................ 458
Tuberculosis, Creosote Treatment of, by Theo. Potter, A.M., M.D...................... 449
Tetanus, Etiology of............ 398
Tetanus, Infectious Origin of.. 190
Thymol Dentifrice............... 510
Theine in the Treatment of Neuralgia........................ 184
Voice in Female Singers Affected by Gynæcological Disorders................ 347
Value of Time, The............ 146
Vegetables, Medical Properties of.......................... 457
Weariness of Dental Practice. 173
Why Carbolic Acid Should Be Discarded by Dentists...... 302
Word Blindness with Unusual Features......................... 340

Waste and Repair................ 196

COLLEGE COMMENCEMENTS.

American College of Dental Surgery........................ 251
Boston Dental College........... 414
Baltimore College of Dental Surgery........................ 358
Chicago College of Dental Surgery............................ 354
Columbian University, Dental Department.................. 255
German American Dental College............................ 360
Harvard University, Dental Department.................. 414
Indiana Dental College......... 361
Kansas City Dental College... 362
Louisville College of Dentistry.......................... 413
Missouri Dental College......... 359
Meharry Dental Department, Central Tennessee College 355
Northwestern University, Dental Department.... ......... 255
Northwestern College of Dental Surgery.................... 357
New York College of Dentistry............................ 252
Ohio College of Dental Surgery........... ................. 251
Philadelphia Dental College.. 254
Pennsylvania College of Dental Surgery.................... 256
Royal College of Dental Surgeons of Ontario............ 355
Southern Medical College, Dental Department......... 352
Tennessee Medical College, Dental Department......... 359
United States Dental College.. 357
University of California, College of Dentistry............ 413
University of Iowa, Dental Department.................. 257
University of Denver, College of Dentistry......... ........ 357
University of Michigan, College of Dental Surgery.... 360
University of Maryland, Dental Department................ 352
University of Pennsylvania, Dental Department,........ 356
Vanderbilt University, Department of Dentistry...... 253
Western Dental College......... 353

EDITORIAL.

Aristol................................ 53
Acid Fruits......................... 108
American Dental Association ..............................364, 464
Acknowledgment.................. 521
Again in the Harness............ 623
Assurance.......................... 211
Barret, Dr. W. C.................. 290
Biographical, Dr. W. H. Atkinson............................ 416
Bibliographical, 54, 55, 160, 523 573, 575, 624..................
Bogus Medical Colleges......... 621
Cleanliness........................ 106
Chancres of the Fingers......... 102
Cleveland, O., Dental Society ..............................366, 521
"Dental Review," The.......... 211
Dental Meeting 5th, 6th, 7th, 8th, District Societies of New York..................... 572
Dental Law.......................... 262
Dental Offices...................... 311
Dental Colleges, Commencements.......................... 415
Fifty Ologies that Concern the Dental Profession........... 203
For Local Anæsthesia........... 207
Faught, Dr. Ashley.............. 212
Indiana State Dental Association.............................. 467
Impacted Third Molar, Case of 50
Instruments......................... 105
Important Announcement, An 264
Mississippi Valley Dental Association....................... 158
Mistakes of Physicians.......... 258
Michigan Meeting................ 313
Mixing Slab, How to Clean.... 207
New Dental Colleges............ 466
New York Dental College...... 212
Office Plans...................... .. 469
Ohio State Dental Meeting.... 522
One More........................... 211
Parts and Percentage............ 260
Pleasant Visit, A.................. 263
Phonograph in Medicine, The 506
Peculiar Case, A.................. 570
Post Graduate Dental Association.............................. 368
Recurring Luxation of the Lower Jaw................ ... 208

Royal College of Surgeons of Dublin, Diplomas Granted by the..................... 208
Specialties in Dental Practice 518
University of Michigan, Dental Department, Requirements for Admission....... 155
University of Michigan, Scheme of Study for the College of Dental Surgery 156
World's Columbian Dental Meeting, The................. 158
Whales and Bacteria............. 159
What Is the Explanation?.... 205

### NOTICES.

American Dental Society...... 363
American Medical Association 201
Committee on Dental Patents 364
Examiners' Meeting.......... .. 524
Illinois State Meeting........... 199
Michigan Dental Association. 363
Missouri State Dental Association........................... 309
Mississippi Valley Dental Association........................ 158
Northern Ohio Dental Association.........................52, 200
National Association of Boards of Dental Examiners...... 308
National Association of Dental Faculties.................. 308
Ohio State Dental Society...... 569
The International Medical Congress at Berlin.......... 192

### OBITUARY.

Bradley, Dr. Calvin.............. 56
Coffin, Dr. C. R.................... 212
Carpenter, Dr. E. R............. 316
Runyan, Dr. Frank C........... 367
Spencer, Mr. F. E................. 316
White, Dr. James W........314, 625

### IN MEMORIAM'

Atkinson, Dr. William H. and Dr. John Stephan......514, 518
White, Dr. James W........... 367

### Northern Ohio Dental Assoiation.

The forty-second annual meeting of this body will be held at Oberlin, O., at the Y. M. C. A. Association Building, Tuesday, May 12th, 1891, at 10 o'clock A. M., continuing three days. A very interesting programme has been prepared, embracing the following subjects, viz:

"Development of the Teeth;" this paper will be presented by Dr. W. H. Whitslar, of Youngstown, the discussion will be opened by Dr. A. J. Dowds, of Canton; second, "The Recurrence of Decay in Teeth," paper by Dr. J. G. Templeton, of Pittsburg, Pa.; third, paper by Dr. W. H. Atkinson, subject, "Hind Sight," discussion opened by Drs. C. R. Butler and E. J. Waye; fourth, "Sanitary Condition of the Mouth, and How Best to Maintain It," paper by Dr. J. F. Dougherty, of Canton, discussion opened by Dr. W. T. Jackman, of Cleveland, and Dr. J. H Wihle, of Canton; there will also be presented a number of voluntary papers, and incidents of office practice, etc.; there will also be several clinics in which the following will be given: "A Tin Filling with Gold Facing," by Dr. S. B. Dewey, of Cleveland; second, "Electricity Supplemented with a paper," by Dr. Frank Craeger, of Fremont, O.; third, "Melting an Ingot of Amalgam," followed by a talk on the same, by Dr. J. F. Siddall, of Oberlin, O.

This programme will be greatly enlarged between this and the time of meeting. It is to be hoped the members will bear this in mind and prepare their papers and matters as promptly as possible, and notify the corresponding secretary. The aim and effort will be to make this one of the largest and best meetings this society has ever held. An earnest invitation is extended to the dental profession throughout the State, and in the adjoining States to be present, and so far as practicable to contribute each his mite to the success of the occasion. Communications in reference to the meeting should be addressed to Dr. Henry Barnes, Cleveland, Ohio.

### Mississippi Valley Dental Association.

The next meeting of this time honored society already promises to be a success. Interesting papers are promised from Cincinnati, Richmond, Wheeling, Chicago, and other cities. The committee are giving the plan adopted last year a thorough trial and so far it works very well.

Any dentist having any thing of interest to show, or to say, are requested to notify the chairman of the executive committee at once, so that every thing may be put in best shape possible.

More definite announcements can be made next month.

J. R. CALLAHAN,
79 W. 8th Street, Cincinnati. Chairman Ex. Com.

Bacteriology Run to Extremes........................................... 45

Improved Hypodermic Syringe............................................ 47

Oliver Wendell Holmes.................................................. 49

To Save the Edge of Sterilized Instruments............................. 50

EDITORIAL.

Case of Impacted Third Molar........................................... 50

Northern Ohio Dental Association....................................... 52

Aristol................................................................ 53

Bibliographical........................................................ 55

Mississippi Valley Dental Association.................................. 55

Obituary—Dr. Bradley................................................... 56

### THE DENTAL REGISTER,

A MONTHLY MAGAZINE DEVOTED TO THE INTERESTS OF THE

**DENTAL PROFESSION.**

*Terms Reduced to* $2.00 *Per Year. Single Copies,* 25 *Cents.*

**EDITED BY PROF. J. TAFT, D.D.S.**

—AND—

**Published by SAM'L A. CROCKER & CO., Ohio Dental and Surgical Depot.**

The volume commences in January and closes in December.

The REGISTER being issued monthly is always fresh, therefore Indispensable to the practitioner who desires to keep posted up with the new inventions and improvements which are daily developed. Neither expense nor effort will be spared to give value and variety to its contents. Articles will be illustrated with well executed engravings whenever they will add to the interest or assist to explanation.

Its extensive circulation and frequency of issue make it one of the BEST DENTAL ADVERTISING MEDIUMS in the United States.

All subscriptions must begin with January or July.

Local or special notices, five lines or less, will be inserted for $3 each insertion; over five lines, 50 cts. per line.

All advertising bills payable in advance, or at furthest, after the issue of the first number containing the advertisement. All transient advertisements to be paid for in advance.

☞CONTRIBUTIONS SOLICITED.

Exclusively Editorial Communications should be addressed to the Editor.

The latest number of the REGISTER may be ordered with or without other goods at any time, and all letters should be addressed to

SAM'L A. CROCKER & CO., Ohio Dental and Surgical Depot, Cincinnati, O.

